# Resilience, Purpose in Life, Loneliness, and Associated Medical Costs in Older Adults

**DOI:** 10.1093/geroni/igab046.688

**Published:** 2021-12-17

**Authors:** Timothy Barnes, Rifky Tkatch, Gandhi Bhattarai, Sandra Kraemer, James Schaeffer, Charlotte Yeh

**Affiliations:** 1 UnitedHealth Group, Minnetonka, Minnesota, United States; 2 UnitedHealth Group, Oak Park, Michigan, United States; 3 OptumLabs, Minnetonka, Minnesota, United States; 4 UnitedHealthcare, Minnetonka, Minnesota, United States; 5 AARP Services, Inc., Washington, District of Columbia, United States

## Abstract

Resilience, purpose in life (PIL), and loneliness have been linked, and used to characterize the health and well-being of older adults. Studies demonstrate that higher resilience, PIL, and minimal loneliness are associated with better late-life outcomes. However, research on how these constructs negatively impact medical costs is limited. Using survey and claims data from a large sample of older adults age 65+ (N=4,496), resilience, PIL, and loneliness were examined to determine associations with medical costs. Among study participants, 11% exhibited low resilience, 19% severe loneliness, and 35% low PIL. Low resilience was associated with 24% higher medical costs compared to participants with high resilience, severe loneliness with 20% higher costs compared to participants with no loneliness, and low PIL marginally associated with 12% higher costs compared to participants with high PIL. Interventions targeting resilience, PIL, and loneliness could be beneficial to promoting successful aging and lowering medical costs.

